# Association Between Postoperative Pain and Inflammatory Biomarkers After Ovariohysterectomy in Dogs: A Pilot Study

**DOI:** 10.1002/vms3.71160

**Published:** 2026-08-10

**Authors:** Alper Yasin Çiplak, Şifanur Aydın, Damla Tuğçe Okur

**Affiliations:** ^1^ Department of Obstetrics and Gynecology Faculty of Veterinary Medicine Ataturk University Erzurum Turkey

**Keywords:** cortisol, C‐reactive protein, dog, inflammatory biomarkers, ovariohysterectomy, pain

## Abstract

**Background:**

Postoperative pain is a complex, multidimensional phenomenon and its relationship with systemic inflammatory and stress‐related biomarkers remains unclear in veterinary patients.

**Objectives:**

To investigate the temporal relationship between postoperative pain intensity and systemic inflammatory or stress‐related biomarkers in dogs undergoing elective ovariohysterectomy (OHE).

**Study Design:**

Prospective clinical study.

**Methods:**

Twenty clinically healthy female dogs were evaluated using a numerical rating scale (0–27) for pain. Serial blood samples were collected at five timepoints: preoperative baseline (*T*_0_), and 1 h (*T*_1_), 3 h (*T*_2_), 6 h (*T*_3_) and Day 7 (*T*_4_) postoperatively. Serum tumour necrosis factor‐α (TNF‐α), interleukin‐6 (IL‐6), haptoglobin (HP), C‐reactive protein (CRP) and cortisol concentrations were measured using canine‐specific ELISA kits. A standardized anaesthetic, surgical and analgesic protocol was applied to all animals. Temporal changes were analysed using the Friedman test, and associations between pain scores and biomarkers were assessed using Spearman's rank correlation.

**Results:**

All variables demonstrated significant time‐dependent changes following OHE (*p* < 0.0001). Pain scores increased in the early postoperative period and returned toward baseline by Day 7. Similarly, TNF‐α, IL‐6, HP, CRP and cortisol showed transient postoperative elevations followed by a decline toward preoperative levels. No statistically significant correlations were detected between pain scores and any biomarker at any timepoint (all *p* > 0.05).

**Conclusions:**

Commonly measured systemic inflammatory and stress‐related biomarkers do not reliably reflect acute postoperative pain intensity in dogs undergoing routine soft tissue surgery. Multimodal assessment approaches integrating validated clinical pain scales with objective parameters are recommended over reliance on biomarker data alone.

## Graphical abstract

Elective ovariohysterectomy in dogs induced significant time‐dependent changes in postoperative pain scores and systemic inflammatory/stress biomarkers. However, these biomarkers did not reliably reflect acute pain intensity, supporting multimodal pain assessment rather than biomarker‐based evaluation alone.

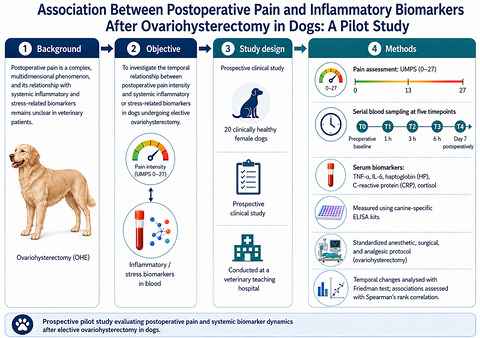

## 1 Introduction

Ovariohysterectomy (OHE) is one of the most frequently performed elective surgical procedures in dogs and remains a standard clinical model for evaluating perioperative pain and tissue injury (Moxon et al. 2025). Despite being a routine intervention, OHE involves traction, manipulation and ligation of the ovarian pedicle, recognized sources of substantial noxious stimulation that can trigger pronounced autonomic, endocrine and behavioural responses (del Mar Granados et al. 2025). This surgical stimulus initiates acute inflammatory and stress pathways, contributing to postoperative discomfort and variation in recovery quality (Flouraki et al. 2024). Accordingly, recent investigations emphasize the importance of optimizing perioperative analgesia and monitoring, as OHE can still elicit measurable physiological and inflammatory alterations even when performed using modern anaesthetic and surgical techniques (Moxon et al. 2025; Dalmolin et al. 2024).

Accurate assessment of postoperative pain in dogs remains challenging because most widely used clinical tools rely on behaviour‐based scoring systems that can be influenced by observer interpretation and situational variability (Salichs et al. 2025). Traditional scales such as numerical rating schemes and composite pain assessments, while commonly applied in clinical practice, exhibit inherent subjectivity and may produce inconsistent results across different observers or clinical environments (Rojsiripornchai et al. 2025). Furthermore, discrepancies frequently arise between subjective pain scores and more objective physiological or mechanical measures, highlighting the limitations of relying solely on clinician‐observed behaviours to quantify nociception (Hölscher et al. 2025). These constraints underscore the need for complementary, biologically grounded indicators that may offer a more objective and reproducible understanding of postoperative pain in dogs.

Pro‐inflammatory cytokines, such as tumour necrosis factor‐α (TNF‐α) and interleukin‐6 (IL‐6), play central roles in the early inflammatory cascade triggered by surgical tissue trauma. TNF‐α typically exhibits a rapid and transient rise within the first hour following an inflammatory insult, whereas IL‐6 increases more gradually and sustains elevated concentrations for several hours, reflecting ongoing cytokine‐mediated acute phase activation (Song et al. 2012). Downstream mediators of this response include acute phase proteins such as haptoglobin (HP) and C‐reactive protein (CRP), both synthesized hepatically under IL‐6 stimulation and recognized as systemic markers of inflammation rather than direct indicators of nociceptive processing (Schmidt and Eckersall 2015). In dogs, elevations in CRP and circulating cytokines have been documented in various inflammatory and postoperative contexts, though their concentrations often display considerable inter‐individual variability and do not consistently parallel clinical pain manifestations (Gommeren et al. 2018). Similar biochemical alterations have been reported following OHE, where surgical manipulation induces measurable shifts in inflammatory and stress‐related biomarkers without a predictable relationship to behavioural pain scores (Kang et al. 2022). Collectively, these findings highlight biological complexity within the postoperative inflammatory response and underscore the need to clarify whether such biomarkers reliably reflect nociceptive experience in dogs.

Growing evidence indicates that nociceptive challenges can trigger measurable systemic inflammatory responses, with acute painful stimuli shown to increase circulating IL‐6 and IL‐1 receptor antagonist concentrations through sympathetic–neuroendocrine activation mechanisms (Griffis et al. 2013). In addition to these short‐term effects, chronic or sustained pain states have been associated with broader immune‐inflammatory alterations: Patients with advanced cancer demonstrate elevated CRP, IL‐6, IL‐18 and soluble TNF receptor‐1 levels, which correlate with symptom severity, including appetite loss, fatigue and global quality‐of‐life impairments (Paulsen et al. 2017). Systematic reviews of musculoskeletal pain further support the involvement of inflammatory mediators, reporting increased CRP, IL‐6 and TNF‐α, along with dysregulated cortisol dynamics suggesting hypothalamic–pituitary–adrenal axis involvement in pain modulation (Sanabria‐Mazo et al. 2022). Despite this growing body of work, limited research has explored these interactions in the context of routine surgical procedures in veterinary medicine. Therefore, the present study aimed to investigate the temporal relationship between postoperative pain and key inflammatory biomarkers, including TNF‐α, IL‐6, IL‐10, cortisol, HP and CRP in dogs undergoing elective OHE, with the objective of determining whether changes in systemic inflammatory mediators reflect or predict the intensity of postoperative pain.

## 2 Materials and Methods

This prospective study was conducted on client‐owned dogs presented for elective OHE at the Atatürk University Veterinary Faculty Animal Hospital. All procedures were performed in accordance with institutional and international guidelines for the care and use of animals in research. Ethical approval for the study protocol was obtained from the Atatürk University Local Ethics Committee for Animal Experiments (Approval No.: 2026/30). A total of 20 clinically healthy female dogs were enrolled after owner informed consent was obtained. Animals were considered suitable for inclusion on the basis of normal physical examination findings and the absence of systemic disease, reproductive pathology or contraindications to anaesthesia or blood sampling. All dogs underwent the same standardized surgical and perioperative management protocols to minimize variability in nociceptive and inflammatory responses.

### 2.1 Anaesthesia and Surgical Procedure

All dogs were fasted for 12 h prior to surgery and underwent a standardized anaesthetic protocol. Premedication consisted of medetomidine hydrochloride at 0.04 mg/kg intramuscularly (Domitor, Zoetis, Turkey) combined with butorphanol at 0.2 mg/kg intramuscularly (Butomidor 10 mg/mL, Richter Pharma, Austria). Anaesthesia was induced with intravenous propofol (2–3 mg/kg, Propofol‐Lipuro, Braun, Germany) to effect and maintained with isoflurane (1%, Isoflurane, Piramal Critical Care Inc., USA) in oxygen delivered via an endotracheal tube. Throughout the procedure, dogs were continuously monitored using a multiparametric system, including heart rate, respiratory rate, non‐invasive blood pressure, oxygen saturation, capnography and electrocardiography to ensure physiologic stability. All animals underwent a routine open midline OHE performed by the same experienced surgeon to minimize procedural variability (Kowaleski et al. 2012). Standardized surgical steps included ventral midline incision, exteriorization of ovaries, pedicle ligation, removal of reproductive tract and routine abdominal closure.

### 2.2 Postoperative Care

Following surgery, all dogs were observed during the early recovery period until complete recovery from anaesthesia was achieved. Animals were discharged only after fulfilling routine clinical discharge criteria, including stable vital parameters, adequate recovery of consciousness and the absence of immediate postoperative complications. Follow‐up examinations were performed on postoperative Days 3 and 7 to evaluate overall clinical condition, surgical wound healing and any procedure‐related complications. Postoperative analgesic management was performed using meloxicam administered subcutaneously at 0.1 mg/kg once daily for two consecutive days, in accordance with routine clinical practice. To avoid interference with early postoperative pain scoring, the initial meloxicam dose was given after completion of the immediate postoperative pain assessments.

### 2.3 Postoperative Pain Assessment and Rescue Analgesia

Postoperative pain was assessed using the University of Melbourne Pain Scale (UMPS), a validated multidimensional composite scoring system for evaluating pain in dogs. The UMPS includes both behavioural and physiological parameters and consists of six categories: physiological data, response to palpation, activity, posture, vocalization and mental status. Each category was scored separately, and the total score was calculated as the sum of the individual category scores, with a maximum possible score of 27.

Pain scoring was performed by a single trained observer who was not involved in the surgical or anaesthetic procedures and was blinded to biomarker results, thereby minimizing observer‐related bias. Assessments were conducted immediately before surgery (*T*_0_, baseline) and at 1 h (*T*_1_), 3 h (*T*_2_), 6 h (*T*_3_) and 7 days (*T*_4_) after OHE.

If the UMPS score exceeded 10/27, rescue analgesia was planned using morphine at 0.3 mg/kg intramuscularly (Morphine Hydrochloride, Galen İlaç, Turkey), in accordance with previously published UMPS‐based postoperative pain assessment recommendations and canine ovariectomy studies using comparable rescue analgesia thresholds (Okur and Polat 2021; Hernandez‐Avalos et al. 2019). All dogs were closely monitored throughout the postoperative assessment period, and rescue analgesia was administered when clinical evaluation indicated inadequate analgesia.

### 2.4 Blood Sampling and Biomarker Analysis

Blood samples for biomarker evaluation were collected at the same defined timepoints as the pain assessments: *T*_0_, *T*_1_, *T*_2_, *T*_3_ and *T*_4_. At each sampling time point, approximately 5 mL of venous blood was collected by the same trained operator via cephalic venipuncture whenever feasible; jugular venipuncture was used only when cephalic access was not suitable. Blood samples were immediately placed into serum collection tubes. The cephalic vein was preferred because it provided reliable peripheral venous access throughout the perioperative period, allowed repeated sampling with minimal interference with the surgical field and airway management and enabled a consistent sampling approach across all time points. After clotting, samples were centrifuged, and serum was aliquoted and stored at −20°C until analysis. Concentrations of TNF‐α, IL‐6, CRP, HP and cortisol were quantified using commercial canine‐specific ELISA kits according to manufacturers’ recommendations. All laboratory staff performing biomarker measurements were blinded to pain score data. Assays were performed in duplicate to ensure analytical precision.

### 2.5 Statistical Analysis

All statistical analyses were performed using IBM SPSS Statistics (Version 29). Descriptive statistics were calculated for all variables at each postoperative timepoint and are presented as mean ± standard deviation (SD). The sample size (*n* = 20) reflected a pragmatic, convenience‐based enrolment of eligible clinical cases over the study period and was considered appropriate for an exploratory analysis of temporal trends and correlations between pain scores and biomarker concentrations. Temporal changes in pain scores and biomarker concentrations (TNF‐α, IL‐6, HP, cortisol and CRP) were evaluated using the Friedman test for repeated measures, given that data did not satisfy parametric assumptions. The relationship between pain scores and biomarker levels was analysed separately for each postoperative timepoint using Spearman's rank correlation coefficient (*ρ*), with corresponding *p* values reported. Correlations could not be evaluated at baseline (*T*_0_) because pain scores were uniformly zero across all subjects. A grayscale heatmap was generated to visualize correlation trends, with each cell displaying the Spearman *ρ* and *p* value. Statistical significance was set at *p* < 0.05 for all analyses.

## 3 Results

No complications were observed in any dog throughout the study period. The mean body weight of the dogs was 25.8 ± 8.1 kg. The mean duration of surgery was 30.9 ± 7.3 min, indicating a relatively consistent operative time across cases.

Significant time‐dependent changes were observed in all measured variables following OHE. Pain scores increased sharply at 1 and 3 h postoperatively and declined thereafter, approaching baseline by Day 7. TNF‐α and IL‐6 concentrations showed parallel early postoperative peaks, whereas HP and CRP exhibited moderate elevations before returning toward baseline. Cortisol increased markedly during the early postoperative period, reaching its highest level at 3 h. Friedman analysis demonstrated that these temporal shifts were statistically significant for all parameters (*p* < 0.0001, Table 1), indicating consistent postoperative alterations in nociceptive, inflammatory and stress‐related responses.

**TABLE 1:** Mean ± standard deviation values of postoperative pain scores and systemic biomarkers at each time point in dogs undergoing ovariohysterectomy.

Parameter	*T*_0_	*T*_1_	*T*_2_	*T*_3_	*T*_4_	*p* value^*^
**Pain score**	0	6.95 ± 1.7	6.65 ± 1.42	5.55 ± 1.43	0.65 ± 0.81	**<0.0001**
**TNF‐α (pg/mL)**	1.39 ± 1	5.21 ± 1.18	5.34 ± 1.79	2.66 ± 1.75	1.11 ± 0.4	**<0.0001**
**IL‐6 (pg/mL)**	43.7 ± 17.9	66.8 ± 26.1	74.5 ± 27.3	62.3 ± 26.15	45 ± 18	**<0.0001**
**Haptoglobin (mg/dL)**	11.3 ± 5.4	18.7 ± 7.4	22.7 ± 10	17.29 ± 8.30	11.2 ± 5.6	**<0.0001**
**Cortisol (ng/mL)**	136 ± 37	223 ± 39.7	298 ± 49.8	200 ± 44.9	129 ± 31	**<0.0001**
**CRP (mg/L)**	2 ± 0.5	3.48 ± 0.99	3.81 ± 0.96	3.01 ± 0.65	2.49 ± 0.32	**<0.0001**

*Note*: T_0_: baseline, immediately before surgery; *T*_1_: 1 h after surgery; *T*_2_: 3 h after surgery; *T*_3_: 6 h after surgery; *T*_4_: 7 days after surgery.

Abbreviations: CRP, C‐reactive protein; IL‐6, interleukin‐6; TNF‐α, tumour necrosis factor‐alpha.

* Indicates the probability that the observed differences occurred by chance. Values <0.05 were considered statistically significant. Bold values indicate statistically significant differences (p < 0.05)

**FIGURE 1:**
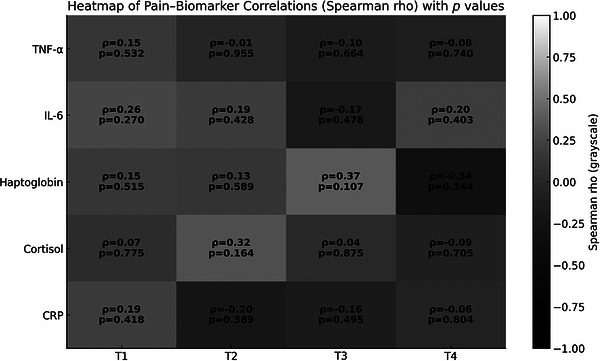
Heatmap illustrating the Spearman correlation coefficients (*ρ*) between postoperative pain scores and inflammatory or stress‐related biomarkers (TNF‐α, IL‐6, HP, cortisol and CRP) at four postoperative timepoints (*T*_1_ = 1 h, *T*_2_ = 3 h, *T*_3_ = 6 h and *T*_4_ = Day 7). Each cell displays the correlation coefficient (*ρ*) and corresponding *p* value. Grayscale intensity reflects the strength and direction of the correlation, with lighter tones indicating more positive associations and darker tones indicating negative associations. No statistically significant correlations were identified at any timepoint (all *p* > 0.05). CRP, C‐reactive protein; IL‐6, interleukin‐6; TNF‐α, tumour necrosis factor‐alpha.

Correlation analysis revealed no significant associations between postoperative pain scores and any of the evaluated biomarkers (TNF‐α, IL‐6, HP, cortisol or CRP) at any of the four postoperative timepoints (all *p* > 0.05). Although weak positive or negative correlation trends were observed at certain intervals, none reached statistical significance. These findings were visualized using a grayscale heatmap in which each cell displays the Spearman correlation coefficient (*ρ*) and corresponding *p* value, confirming the absence of meaningful relationships between nociceptive responses and inflammatory or stress‐related biomarker dynamics following OHE (Figure 1).

## 4 Discussion

In this prospective clinical study, postoperative nociceptive, inflammatory and stress‐related responses were characterized in dogs undergoing elective OHE by monitoring behavioural pain scores alongside circulating TNF‐α, IL‐6, HP, CRP and cortisol at defined timepoints. As expected, surgery was well tolerated, with no perioperative complications and relatively uniform operative times across patients. All measured variables showed significant time‐dependent changes: Pain scores rose markedly in the early postoperative period and approached baseline values by Day 7, whereas pro‐inflammatory cytokines and acute phase proteins demonstrated transient postoperative increases before declining toward preoperative levels, and cortisol exhibited a pronounced early peak. However, despite these coordinated temporal shifts, no statistically significant associations were identified between pain scores and any of the evaluated biomarkers at any postoperative timepoint, indicating that the hypothesized parallel elevation of nociceptive scores and systemic inflammatory or stress markers was not supported by the present data.

In the present study, OHE induced clear time‐dependent changes in nociceptive, inflammatory and stress‐related variables, with early postoperative increases in pain scores, TNF‐α, IL‐6, HP, CRP and cortisol followed by a gradual return toward baseline values. This overall pattern is consistent with previous work showing that elective ovariectomy or OHE in healthy bitches elicits a measurable acute‐phase response, including postoperative rises in CRP and other acute phase proteins that mirror the magnitude of surgical trauma (Del Romero et al. 2020). Similarly, studies of bitches undergoing OHE or other abdominal procedures have documented postoperative increases in IL‐6 and IL‐10, typically peaking within the first few days after surgery and declining thereafter, supporting the concept that surgical manipulation provokes a transient cytokine surge rather than sustained systemic inflammation (Dalmolin et al. 2024; Dąbrowski et al. 2015). Postoperative cortisol dynamics observed in our dogs, with an early peak within hours of surgery, also align with earlier reports in which OHE and related procedures led to significant, but short‐lived, elevations in plasma cortisol as an indicator of surgical stress (Fox et al. 1994). Importantly, the absence of perioperative complications and the relatively uniform surgical times in our population are consistent with the moderate magnitude and transient nature of the biomarker changes, in agreement with studies suggesting that exaggerated or prolonged CRP responses are more often associated with postoperative complications or more extensive tissue trauma (Gommeren et al. 2018; Michelsen et al. 2012).

Despite these coordinated temporal shifts, we did not detect significant correlations between behavioural pain scores and any of the measured biomarkers at individual postoperative timepoints, a finding that underscores the complex and only partially overlapping nature of nociceptive and inflammatory pathways. Similar dissociation has been noted in canine studies in which postoperative CRP or cytokine concentrations changed significantly after OHE yet did not consistently parallel clinical pain assessments or behavioural indicators of discomfort (Schmidt and Eckersall 2015; Schmidt et al. 2018). In human surgical populations, some investigations have reported associations between higher IL‐6 or CRP concentrations and greater postoperative pain or slower recovery, whereas others found only weak or inconsistent relationships, suggesting that systemic inflammatory markers capture tissue injury and immune activation rather than subjective pain intensity in essence (Cocea and Stoica 2024; Repo et al. 2019). Acute phase proteins, such as CRP and HP, are strongly driven by hepatic responses to cytokines and may peak at different times than behavioural pain, whereas cortisol is influenced by multiple stressors, including hospitalization, handling and individual temperament (Dalmolin et al. 2024).

This study has several limitations that should be considered when interpreting the findings. The sample size was relatively small and drawn from a single institution, which may limit the generalizability of the results to broader clinical populations and to dogs undergoing different types or grades of surgical trauma. Only clinically healthy, intact female dogs undergoing elective OHE were included, so the observed patterns of pain and biomarker responses may not reflect those in animals with comorbidities, emergency procedures or more extensive soft tissue or orthopaedic surgeries. Pain was assessed using a numerical rating scale, which, although practical in a clinical setting, is inherently subjective and may be less sensitive or specific than fully validated composite pain scales. In addition, standard perioperative and postoperative analgesia was administered to all dogs, and potential variation in analgesic effect may have influenced both pain scores and biomarker dynamics in ways that could not be fully controlled. A further limitation of the present study is the potential confounding effect of rescue analgesia on the relationship between postoperative pain scores and systemic biomarkers. For ethical reasons, rescue analgesia was planned and administered when pain exceeded the predefined threshold or when clinical assessment indicated inadequate analgesia. Although this approach was necessary to ensure animal welfare, it may have attenuated postoperative pain scores and reduced the observable variability in pain intensity. Consequently, the correlation analyses between pain scores and biomarkers should be interpreted as reflecting associations primarily within the range of pain below the rescue analgesia threshold. Therefore, the present study may not fully capture biomarker responses associated with higher levels of postoperative pain.

The biomarker panel was limited to selected systemic mediators and was measured at discrete timepoints; more frequent sampling, inclusion of additional inflammatory or neuroendocrine markers and assessment of local tissue or cerebrospinal changes might have provided a more detailed characterization of the nociceptive‐inflammatory interface. Finally, the study was exploratory in nature and may have been underpowered to detect modest correlations between pain scores and biomarker concentrations, particularly given the biological variability inherent to both measures.

In this prospective cohort of dogs undergoing elective OHE, surgery was associated with clear, time‐dependent changes in behavioural pain scores and in circulating concentrations of TNF‐α, IL‐6, HP, CRP and cortisol, consistent with a coordinated nociceptive, inflammatory and stress response to routine soft tissue trauma. However, no statistically significant associations were identified between postoperative pain scores and any of the evaluated systemic biomarkers at individual timepoints. These findings suggest that, under the conditions of this study, commonly measured inflammatory and stress‐related markers may not serve as reliable stand‐alone surrogates for acute postoperative pain intensity in dogs. Instead, they support the continued use of multimodal pain assessment strategies in which clinical scoring systems are complemented—but not replaced—by selected objective measurements. Future studies involving larger and more diverse populations, additional biomarker candidates and alternative objective methods of nociceptive assessment may help clarify the circumstances under which biochemical indicators can meaningfully contribute to postoperative pain evaluation in veterinary patients.

## 5 Conclusion

In conclusion, this study clearly demonstrated that elective OHE induces pronounced, time‐dependent nociceptive, inflammatory and stress responses in dogs. Although parallel increases were observed in postoperative pain scores and circulating concentrations of TNF‐α, IL‐6, HP, CRP and cortisol during the early postoperative period, no statistically significant associations were identified between these variables. This finding indicates that systemic biomarkers primarily reflect tissue injury and immune activation rather than directly representing the intensity of clinical pain. Therefore, reliance on biomarkers alone appears insufficient for clinical decision‐making, and their use should be integrated with behavioural and clinical pain assessment tools. In addition, potential influences such as individual variability, analgesic protocols and environmental stressors should be considered when interpreting biomarker dynamics. Future studies with larger sample sizes and more robust methodological designs are warranted to clarify under which conditions biochemical indicators may meaningfully contribute to postoperative pain assessment in veterinary patients.

## Author Contributions

**Alper Yasin Çiplak**: conceptualization, methodology, formal analysis, funding, investigation, writing – original draft preparation, writing – review and editing. **Şifanur Aydın**: methodology, investigation, formal analysis. **Damla Tuğçe Okur**: conceptualization, methodology, formal analysis, funding, investigation, writing – original draft preparation, writing – review and editing

## Funding

The authors have nothing to report.

## Ethics Statement

This prospective study was approved by the Atatürk University Local Board of Ethics Committee (04/2026).

## Conflicts of Interest

The authors declare no conflicts of interest.

## Data Availability

Data sets and analyses are available from the corresponding author upon reasonable request.
